# Supplementary material for: Identification of photoperiod-induced specific miRNAs in the adrenal glands of Sunite sheep (Ovis aries)
Source: Front Vet Sci. 2022 Jul 22;9:888207. doi: 10.3389/fvets.2022.888207 (PMC9354845; doi:10.3389/fvets.2022.888207)
Supplement: Supplementary file 1 [file Data_Sheet_1.ZIP › Supplementary file/miRDeep2 file.zip!/miRDeep2 file/Know_Structure/SPLP42A3/oar-mir-377.pdf]

miRBase precursor : oar-mir-377
Total read count : 36
oar-miR-377-5p read count : 10
oar-miR-377-3p read count : 15
remaining reads : 11

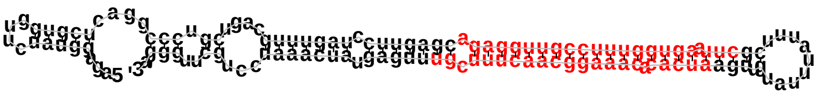

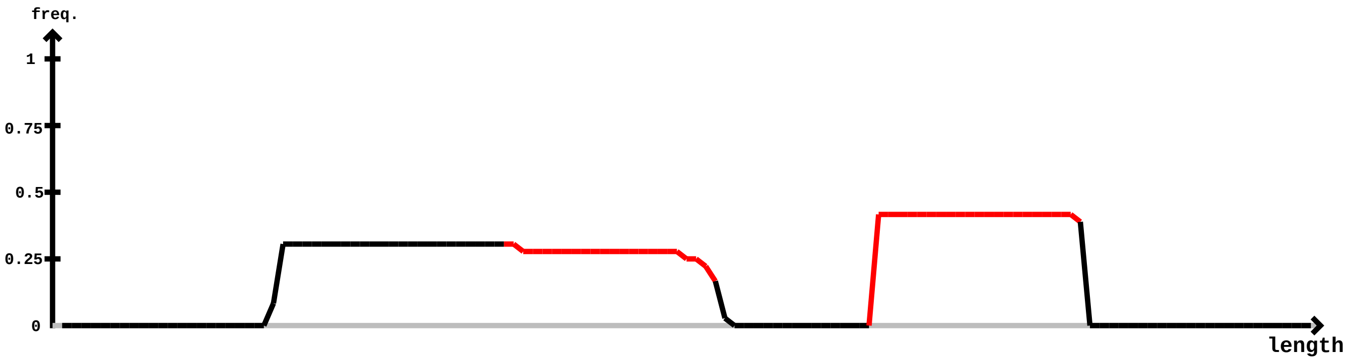


```
                                                                                     oar-miR-377-3p
                                              oar-miR-377-5p
5'- agagguaucuuggugcucaggcccugcugacguuugauccuugagcagagguugccuuuggugaauucgcuuuauuuauguugaaucacacaaaggcaacuuucguuugaguaucaaauccugcuuggga   -3'   exp
    ...(((((....)))))....(((.((....(((((((.(((((((.(((((((((((((((((.(((((.........).)))))))).))))))))))))).))))))).)))))))...))..))).    reads   mm
    .....................cccugcugacguuugauccuugagc....................................................................................    3       0
    ......................ccugcugacguuugauccuugagc....................................................................................    7       0
    ......................ccugcugacguuugauccuugagca...................................................................................    1       0
    ..............................................agagguugccuuuggugа..................................................................    1       0
    ..............................................agagguugccuuuggugaau................................................................    1       0
    ..............................................agagguugccuuuggugaauu...............................................................    2       0
    ..............................................agagguugccuuuggugaauuc..............................................................    5       0
    ..............................................agagguugccuuuggugaauucg.............................................................    1       0
    ....................................................................................aucacacaaaggcaacuuucg.........................    1       0
    ....................................................................................aucacacaaaggcaacuuucgu........................    14      0
```
